# Supplementary material for: Navoximod modulates local HSV-1 replication to reshape tumor immune microenvironment for enhanced immunotherapy via an injectable hydrogel
Source: Commun Biol. 2023 Jun 9;6:621. doi: 10.1038/s42003-023-04983-z (PMC10256817; doi:10.1038/s42003-023-04983-z)
Supplement: Supplementary file 2 — Supplementary Information [file 42003_2023_4983_MOESM2_ESM.pdf]

Supplementary Information

Navoximod modulates local HSV-1 replication to reshape tumor immune microenvironment for enhanced immunotherapy via an injectable hydrogel

Qiuyu Zhuang[1, 2]†, Binyu Zhao[1]†, Zhiwen Lin[1], Yuzhi Liang[1], Qingfu Zhao[1], Yunhao Wang[1], Naishun Liao[1], Haibin Tu[1], Youshi Zheng[1, 2], Hengkai Chen[4], Yongyi Zeng[1, 4], Da Zhang[1, 2]*, Xiaolong Liu[1, 2, 3]*

[1]The United Innovation of Mengchao Hepatobiliary Technology Key Laboratory of Fujian Province, Mengchao Hepatobiliary Hospital of Fujian Medical University, Fuzhou 350025, P. R. China; The Liver Center of Fujian Province, Fujian Medical University, Fuzhou 350025, P. R. China

[2]Mengchao Med-X Center, Fuzhou University, Fuzhou 350116, P. R. China

[3]CAS Key Laboratory of Design and Assembly of Functional Nanostructures, Fujian Institute of Research on the Structure of Matter, Chinese Academy of Sciences, Fuzhou 350002, P. R. China

[4]The First Affiliated Hospital of Fujian Medical University, Fuzhou 350025, P. R. China

*Corresponding authors: Da Zhang (zdluoman1987@163.com); Xiaolong Liu (xiaoloong.liu@gmail.com)

†These authors contributed equally.

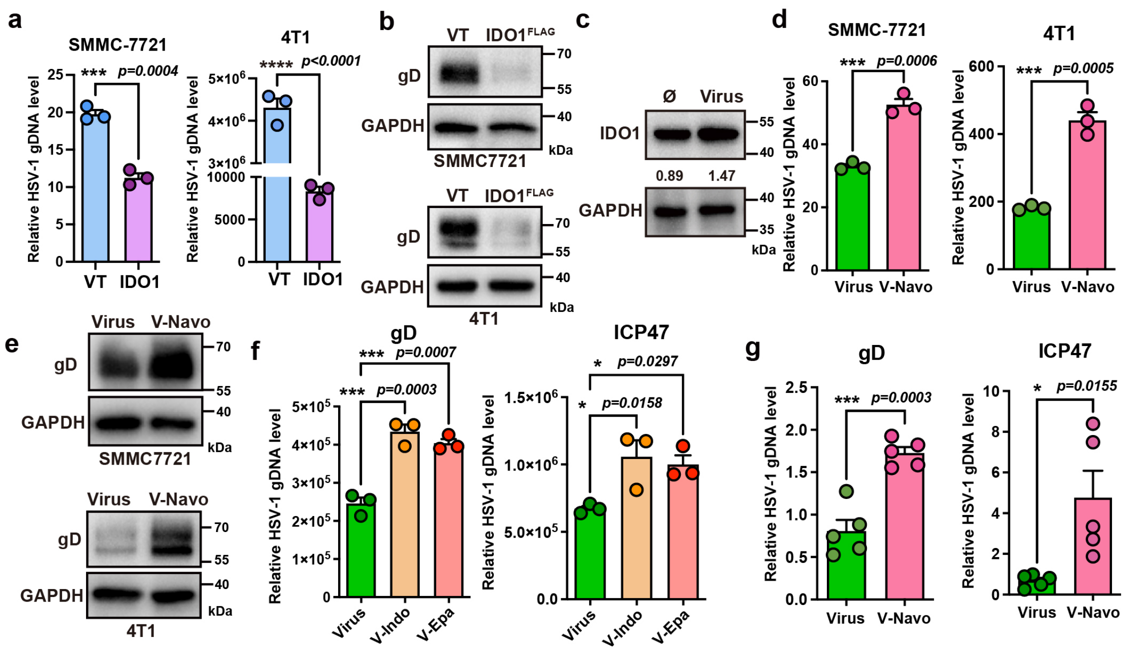

Supplementary Fig. 1 The combination effect of HSV-1 and IDO1 inhibitor Navoximod.

(a and b) SMMC-7721 or 4T1 cells transfected with empty vector (VT) or IDO1 construct were infected with HSV-1 for 24 h. **(a)** RT-qPCR analysis of gD DNA level (n=3; Data were shown as means ± SEM). **(b)** Western blotting analysis of gD and GAPDH. **(c)** Hepa1-6 cells infected with 20 MOI HSV-1 for 24 h were analyzed by western blotting for IDO1 expression. GAPDH was used as a loading control. The gray value ratios of IDO1/GAPDH were shown. **(d and e)** SMMC-7721 or 4T1 cells were treated with 5 MOI HSV-1 or 5 MOI HSV-1 plus 1 μM Navoximod (V-Navo). **(d)** RT-qPCR analysis of gD DNA level (n=3; Data were shown as means ± SEM). **(e)** Western blotting analysis of gD and GAPDH. **(f)** RT-qPCR analysis of gD (left) and ICP47 (right) DNA level in Hepa1-6 cells treated with 5 MOI HSV-1, 5 MOI HSV-1 plus 1 μM Indoximod (V-Indo) or 5 MOI HSV-1 plus 250 μM Epacadostat (V-Epa) for 24 hours (n=3; Data were shown as means ± SEM). **(g)** RT-qPCR analysis of the intratumoral HSV-1 genomic gD (left) and ICP47 (right) DNA level in 4T1 tumor model 8 days after the indicated treatments (n=5; Data were shown as means ± SEM).

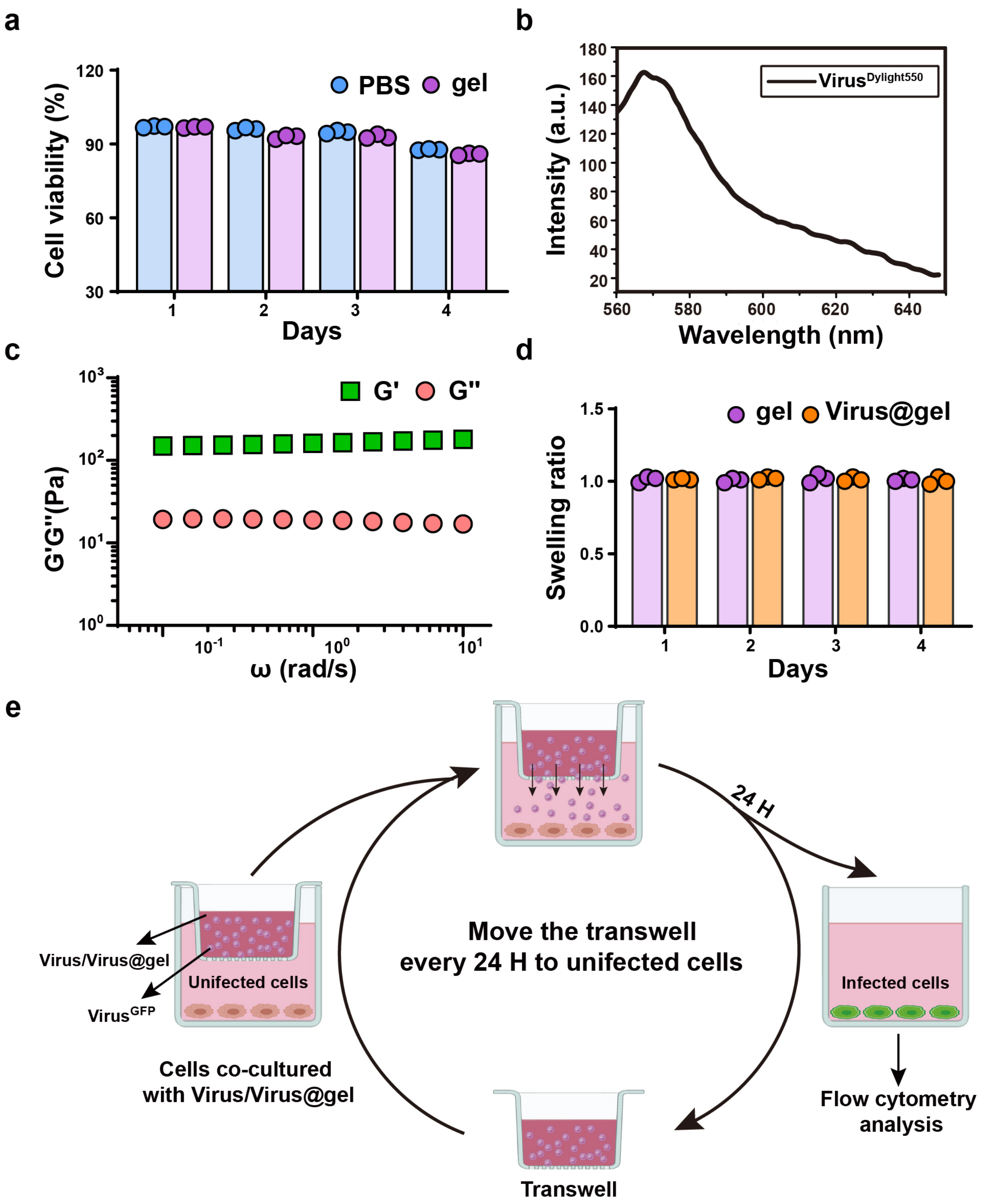

Supplementary Fig. 2 Preparation and characterization of HSV-1-based silk-gels (Virus@gel). (a) The cell viability of indicated groups was determined by flow cytometry analysis using Annexin V-APC and PI staining (n=3). **(b)** Fluorescence spectra of HSV-$1^{\text{Dylight 550}}$ at 520nm excitation for **Fig. 3d and 3h**. **(c)** The storage modulus (G') and loss modulus (G'') of Virus@gel at appropriate strain. **(d)** The swelling ratio of silk-hydrogel and Virus@gel (n=3). **(e)** The experimental procedure

of sustained release assay for **Fig. 3e and 3f**.

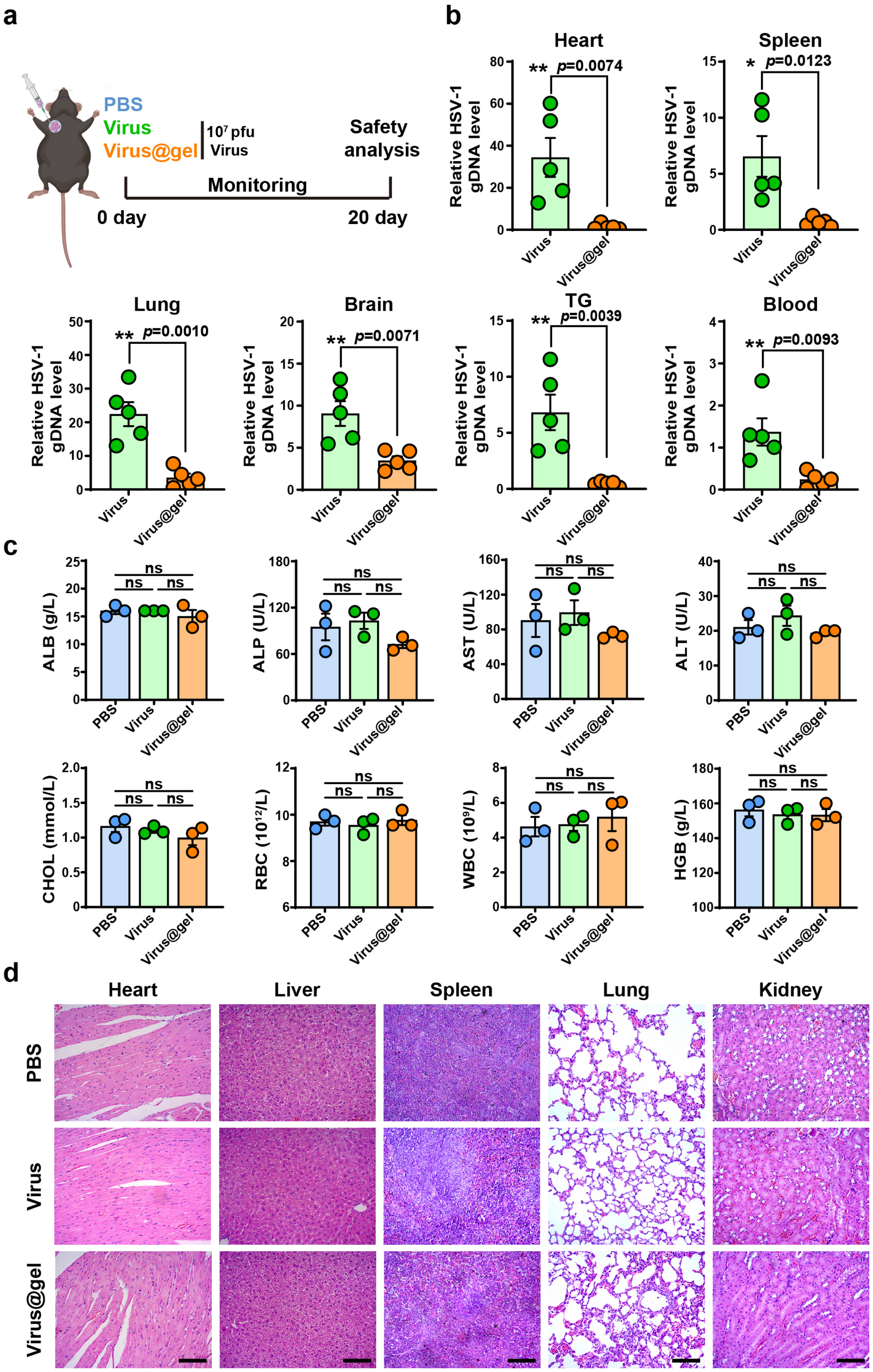

a
PBS
Virus
Virus@gel
10^7 pfu Virus
Safety analysis
Monitoring
0 day
20 day
b
Heart
Spleen
Lung
Brain
TG
Blood
Relative HSV-1 gDNA level
p=0.0074
p=0.0123
p=0.0010
p=0.0071
p=0.0039
p=0.0093
Virus
Virus@gel
c
ALB (g/L)
ALP (U/L)
AST (U/L)
ALT (U/L)
CHOL (mmol/L)
RBC (10^12/L)
WBC (10^9/L)
HGB (g/L)
ns
PBS
Virus
Virus@gel
d
Heart
Liver
Spleen
Lung
Kidney
PBS
Virus
Virus@gel

Supplementary Fig. 3 Biosafety of Virus@gel after subcutaneous administration.

(a) Schematic illustration of the biosafety evaluation of Virus@gel. **(b)** HSV-1 genomic DNA levels, which were represented by HSV-1 gD DNA level in the indicated organs, were measured by RT-qPCR analysis 20 days after indicated treatments (n=5; Data were shown as means ± SEM). **(c)** Blood biochemistry and routine indexes of the C57BL/6 mice 20 days after indicated treatments (n=3; Data were shown as means ± SEM). **(d)** Histological H&E staining of major organs 20 days after indicated treatments. Scale bar: 100 μm.

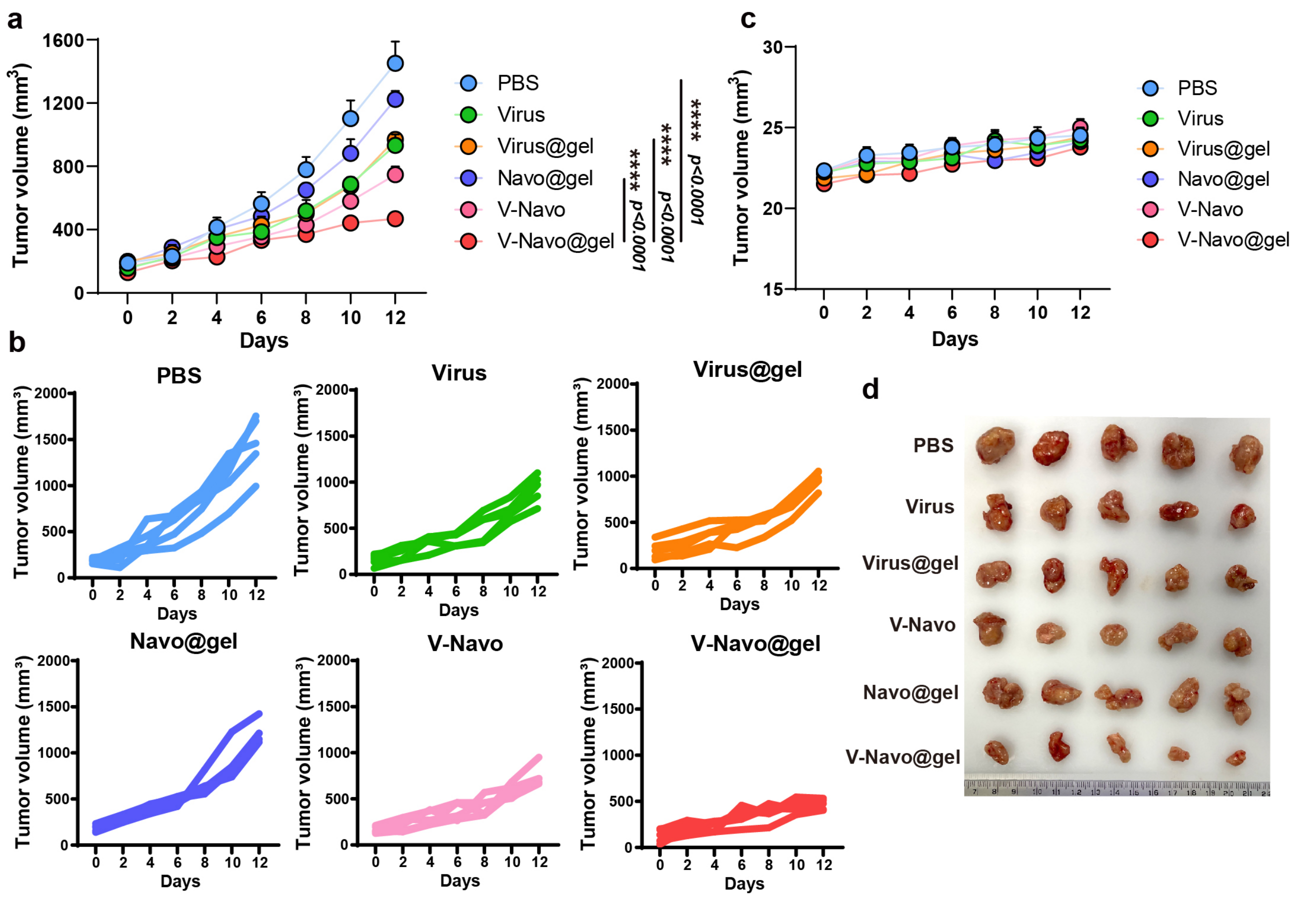

Supplementary Fig. 4 ***In vivo*** **elimination of subcutaneous 4T1 tumors by HSV-1 and Navoximod loaded silk-hydrogels (V-Navo@gel).**

(a and b) Tumor volumes of mice with indicated treatments. (n=5; Data were shown as means ± SEM). **(c)** Inoculated mice weight changes during the whole measurement (n=5; Data were shown as means ± SEM). **(d)** Tumor images isolated from mice after the indicated treatments.

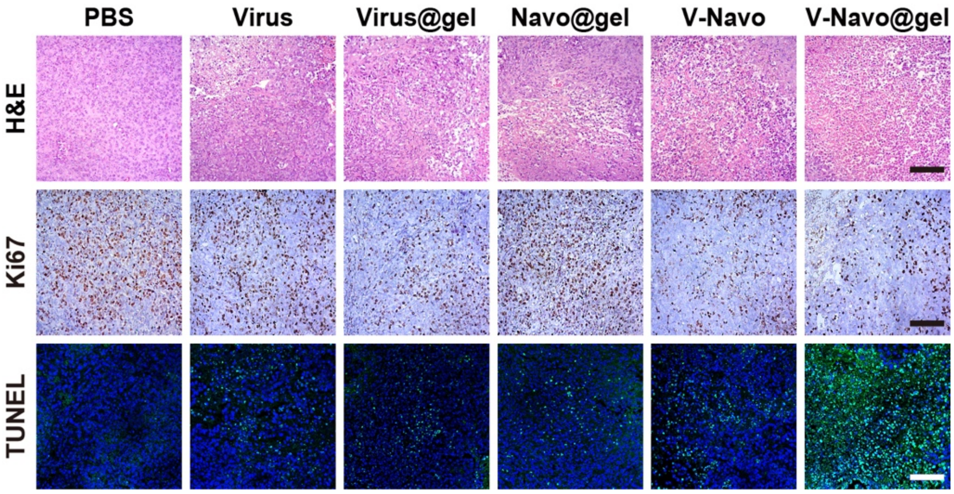

Supplementary Fig. 5 Representative images of tumor slices stained with H&E, Ki67 and TUNEL after indicated treatments. Scale bar: 100 μm.

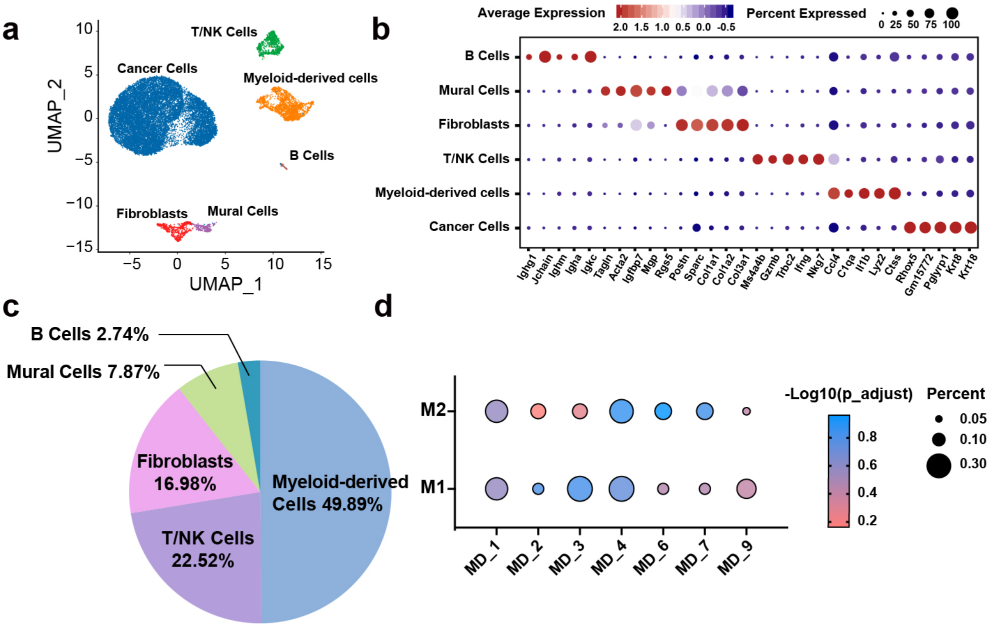

Supplementary Fig. 6 Identification of cell subsets in tumor tissues after indicated treatments. **(a)** UMAP plot showing all identified cell populations within the scRNA-seq experiment. **(b)** Bubble heatmap showing expression of selected signature genes in each cluster. Bubble size represents the percentage of expressing cells, colored based on normalized expression levels. **(c)** Population percentages of the indicated cell subsets. **(d)** Gene enrichment for classical cell type M1 and M2 in comparisons within the identified macrophage clusters. The bubble size represents the proportion of upregulated genes of clusters based on the gene signature on the y axis. The color represents p value.

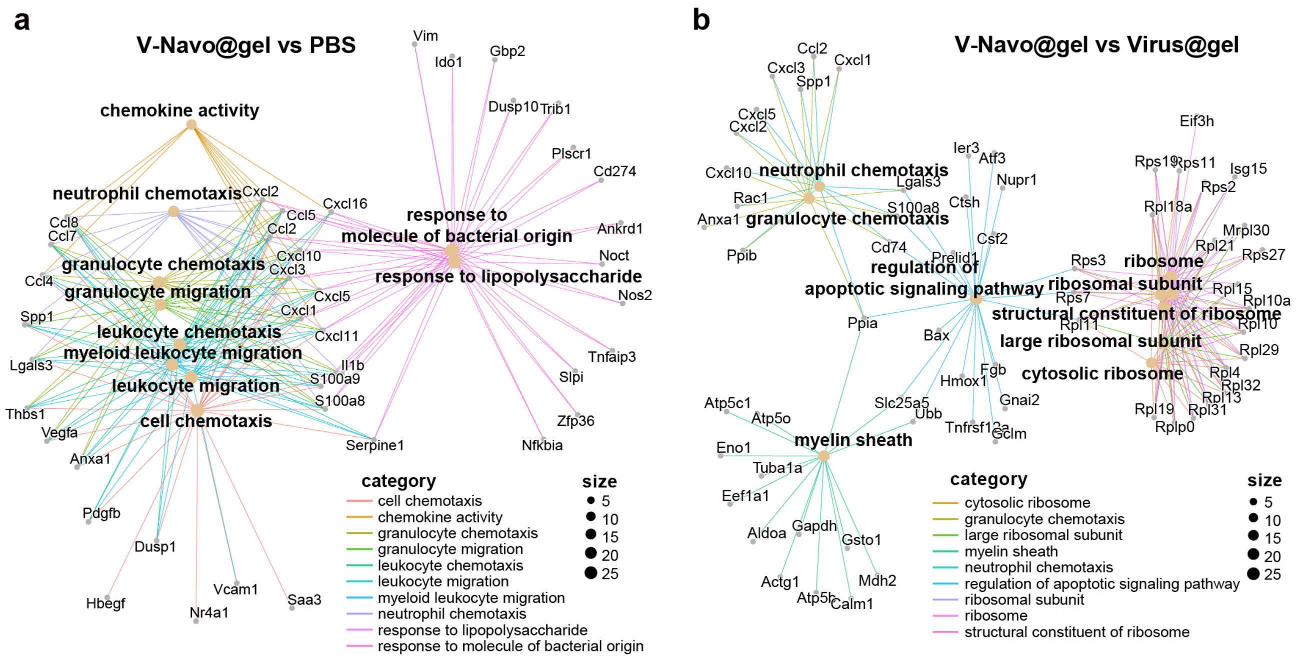

Supplementary Fig. 7 The enriched pathways with differently expressed genes in V-Navo@gel versus PBS group **(a)** or V-Navo@gel versus Virus@gel group **(b)**.

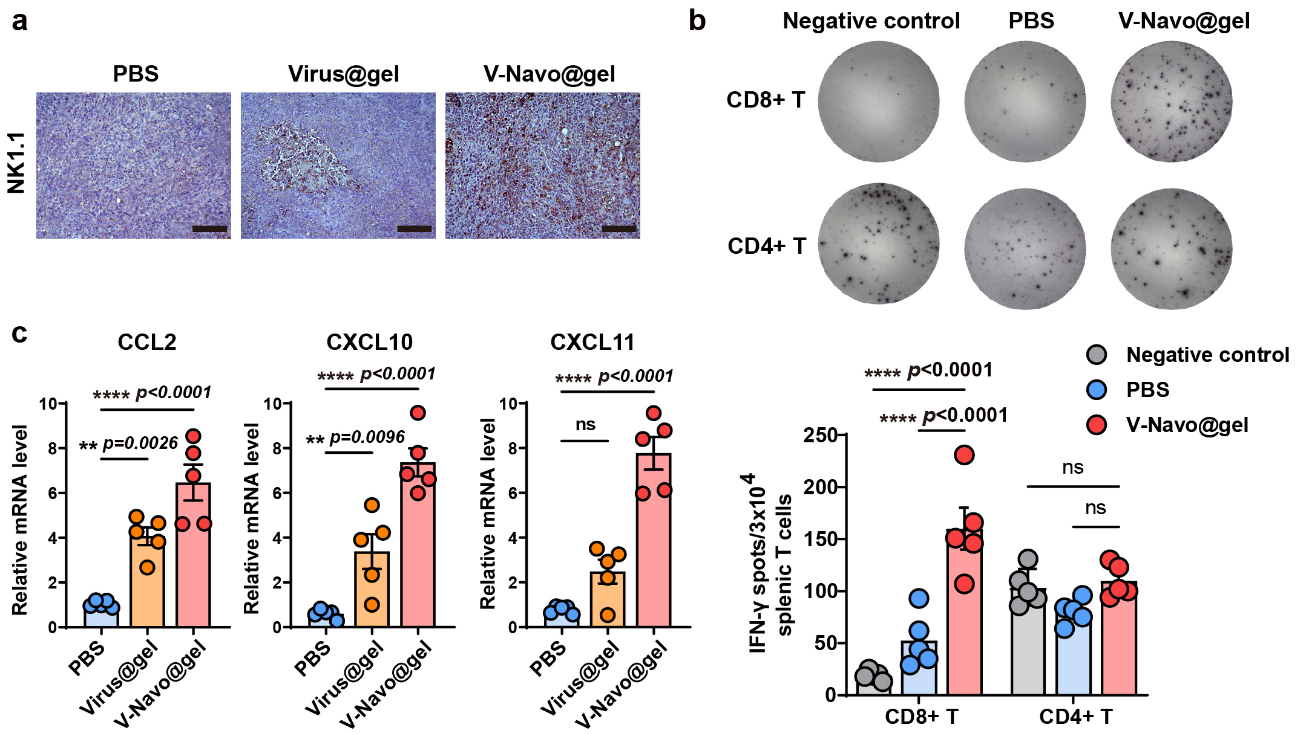

Supplementary Fig. 8 ***In vivo*** **modulation of antitumor immune responses by V-Navo@gel.**

(a) Representative images of tumor slices stained with NK1.1 after indicated treatments. Scale bar: 100 μm. **(b)** Representative images (up) and analysis (bottom) of the tumor specific T cell with IFN-γ ELISPOT assay (n=5; Data were shown as means ± SEM). **(c)** RT-qPCR analysis of the intratumoral CCL2, CXCL10 and CXCL11 expression 8 days after the indicated treatments (n=5; Data were shown as means ± SEM).

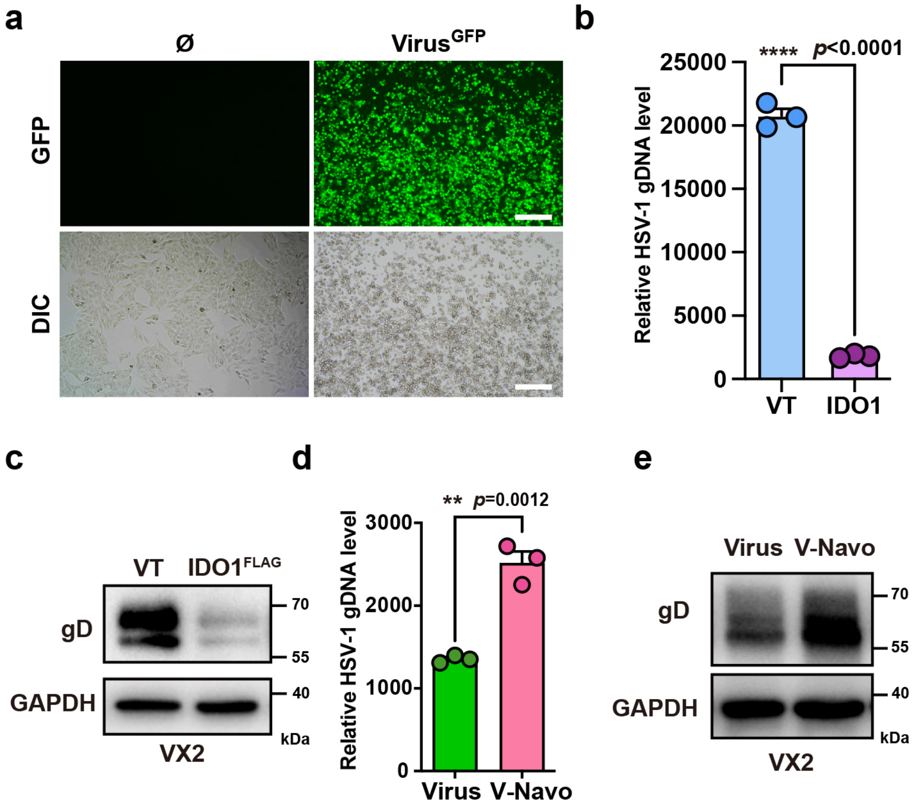

Supplementary Fig. 9 (a) Representative fluorescence imaging of HSV-1 to kill VX-2 tumor cells *in vitro*. Scale bar: 200 μm. **(b and c)** VX-2 cells transfected with empty vector (VT) or Flag-tagged IDO1 construct were infected with HSV-1 for 24 h. **(b)** RT-qPCR analysis of gD DNA level (n=3; Data were shown as means ± SEM). **(c)** Western blotting analysis of gD and GAPDH. **(d and e)** VX-2 cells were treated with 5 MOI HSV-1 or 5 MOI HSV-1 plus 1 μM Navoximod (V-Navo). **(d)** RT-qPCR analysis of gD DNA level (n=3; Data were shown as means ± SEM). **(e)** Western blotting analysis of gD and GAPDH.

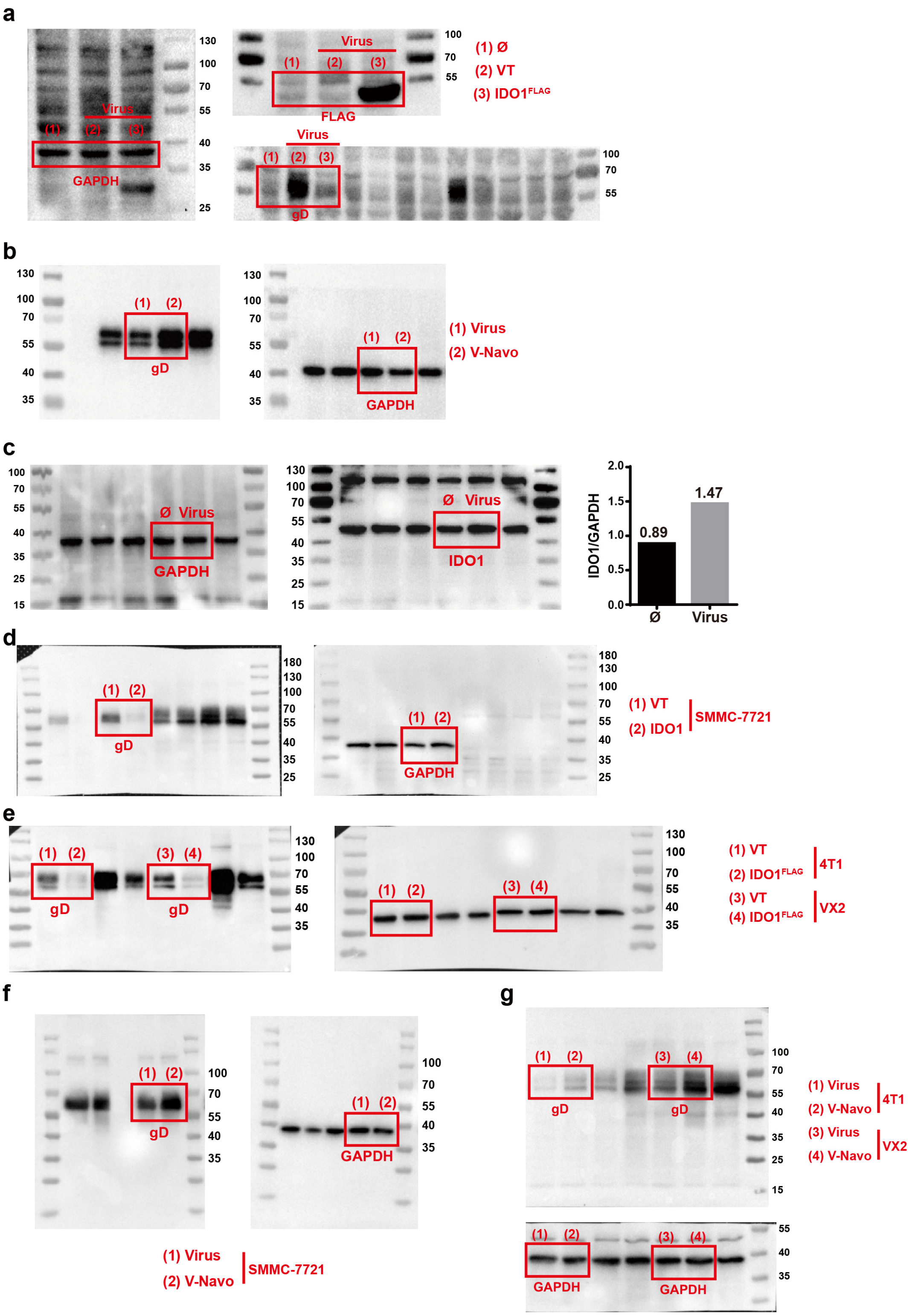

Supplementary Fig. 10 Images of original uncropped western blots. The red rectangles outline the images used in the listed figures.

(**a**) Full scan for Fig. 2a. (**b**) Full scan for Fig. 2g. (**c**) Full scan for Supplementary Fig. 1c. The gray value ratios of IDO1/GAPDH were shown. (**d and e**) Full scan for Supplementary Fig. 1b and Supplementary Fig. 9c. (**f and g**) Full scan for Supplementary Fig. 1e and Supplementary Fig. 9e.

a

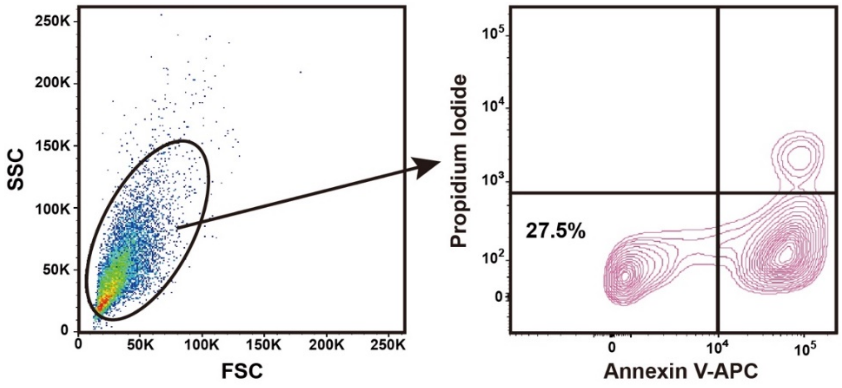

SSC
FSC
250K
200K
150K
100K
50K
0
50K
100K
150K
200K
250K
Propidium Iodide
Annexin V-APC
27.5%

b

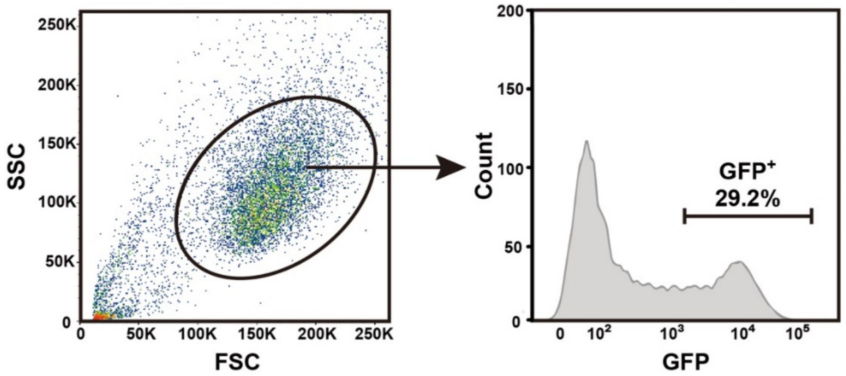

SSC
FSC
Count
GFP
GFP+
29.2%

c

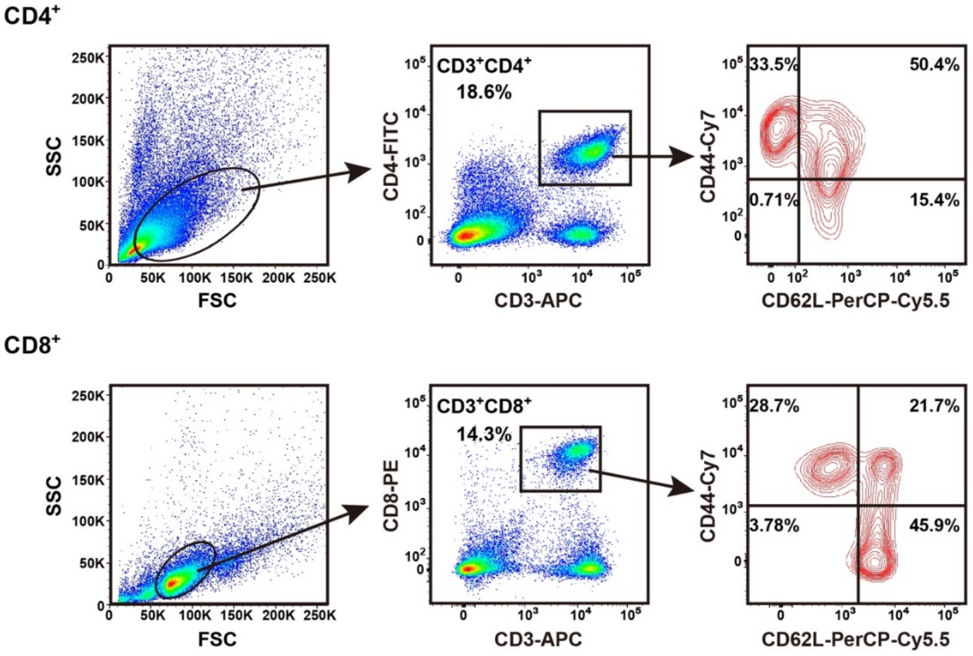

CD4+
SSC
FSC
CD4-FITC
CD3-APC
CD3+CD4+
18.6%
CD44-Cy7
CD62L-PerCP-Cy5.5
33.5%
50.4%
0.71%
15.4%
CD8+
CD8-PE
CD3+CD8+
14.3%
28.7%
21.7%
3.78%
45.9%

d

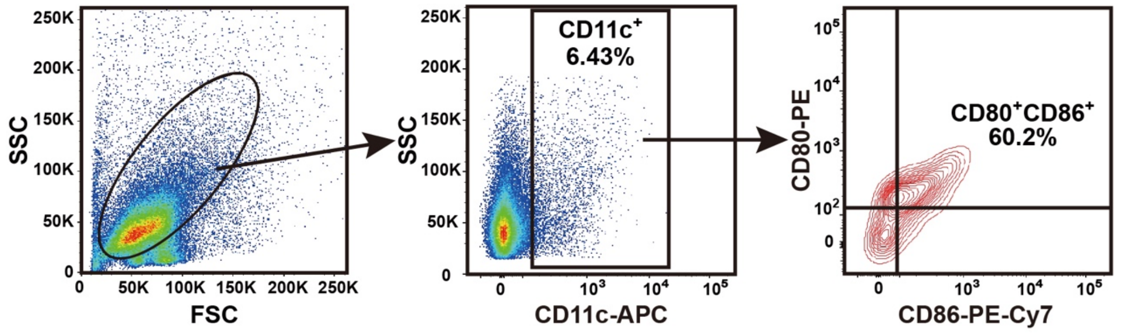

e

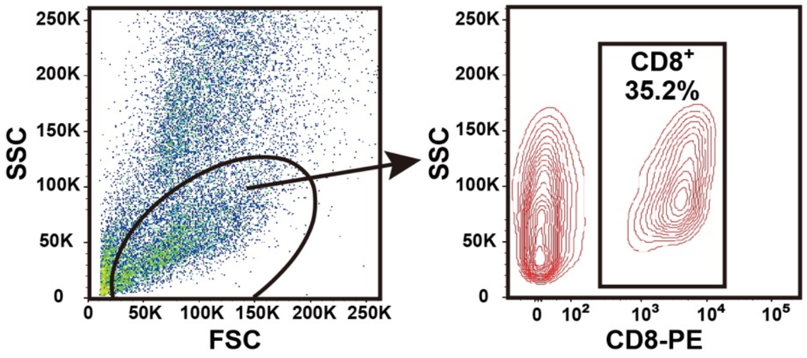

f

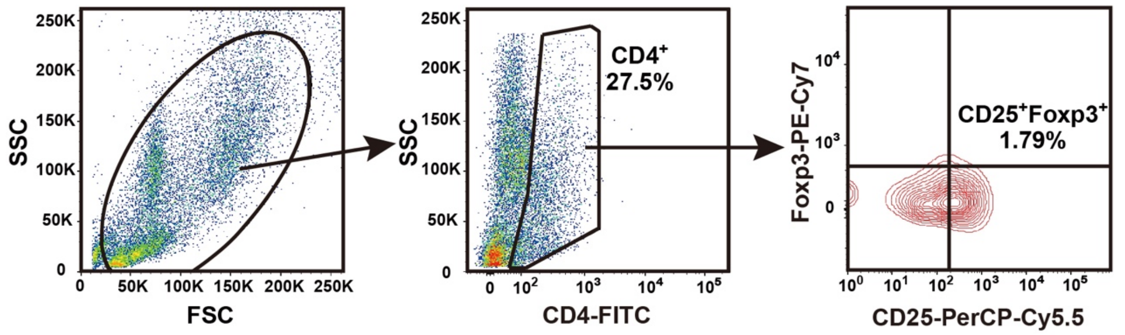

Supplementary Fig. 11 Gating strategies used for the indicated figures.

(**a**) Gating strategy for Fig. 2h. (**b**) Gating strategy for Fig. 3e. (**c**) Gating strategy for Fig. 4k. (**d**) Gating strategy for Fig. 7b. (**e**) Gating strategy for Fig. 7c. (**f**) Gating strategy for Fig. 7e.
