# Supplementary material for: Navoximod modulates local HSV-1 replication to reshape tumor immune microenvironment for enhanced immunotherapy via an injectable hydrogel
Source: Commun Biol. 2023 Jun 9;6:621. doi: 10.1038/s42003-023-04983-z (PMC10256817; doi:10.1038/s42003-023-04983-z)
Supplement: Supplementary file 5 — Reporting Summary [file 42003_2023_4983_MOESM5_ESM.pdf]

nature portfolio

Corresponding author(s): Xiaolong Liu

Last updated by author(s): May 17, 2023

Reporting Summary

Nature Portfolio wishes to improve the reproducibility of the work that we publish. This form provides structure for consistency and transparency in reporting. For further information on Nature Portfolio policies, see our Editorial Policies and the Editorial Policy Checklist.

Statistics

For all statistical analyses, confirm that the following items are present in the figure legend, table legend, main text, or Methods section.

n/a	Confirmed	
☐	☒	The exact sample size (*n*) for each experimental group/condition, given as a discrete number and unit of measurement
☐	☒	A statement on whether measurements were taken from distinct samples or whether the same sample was measured repeatedly
☐	☒	The statistical test(s) used AND whether they are one- or two-sided<br>*Only common tests should be described solely by name; describe more complex techniques in the Methods section.*
☐	☒	A description of all covariates tested
☐	☒	A description of any assumptions or corrections, such as tests of normality and adjustment for multiple comparisons
☐	☒	A full description of the statistical parameters including central tendency (e.g. means) or other basic estimates (e.g. regression coefficient) AND variation (e.g. standard deviation) or associated estimates of uncertainty (e.g. confidence intervals)
☐	☒	For null hypothesis testing, the test statistic (e.g. *F*, *t*, *r*) with confidence intervals, effect sizes, degrees of freedom and *P* value noted<br>*Give P values as exact values whenever suitable.*
☒	☐	For Bayesian analysis, information on the choice of priors and Markov chain Monte Carlo settings
☒	☐	For hierarchical and complex designs, identification of the appropriate level for tests and full reporting of outcomes
☒	☐	Estimates of effect sizes (e.g. Cohen's *d*, Pearson's *r*), indicating how they were calculated

Our web collection on statistics for biologists contains articles on many of the points above.

Software and code

Policy information about availability of computer code

Data collection	No software was used.
Data analysis	Graphpad Prism 9, FlowJo, Seurat (3.1.2), Cellphone DB (2.1.0), Zen 2012

For manuscripts utilizing custom algorithms or software that are central to the research but not yet described in published literature, software must be made available to editors and reviewers. We strongly encourage code deposition in a community repository (e.g. GitHub). See the Nature Portfolio guidelines for submitting code & software for further information.

Data

Policy information about availability of data

All manuscripts must include a data availability statement. This statement should provide the following information, where applicable:

- Accession codes, unique identifiers, or web links for publicly available datasets
- A description of any restrictions on data availability
- For clinical datasets or third party data, please ensure that the statement adheres to our policy

RNA-seq data were deposited into Genome Sequencing Achieve for Human database (https://ngdc.cncb.ac.cn/gsa-human/) under the accession number of HRA000464. ScRNA-seq data were deposited into Genome Sequencing Achieve database (https://hgdc.cncb.ac.cn/gsa) under the accession number of CRA008926.

Human research participants

Policy information about studies involving human research participants and Sex and Gender in Research.

Reporting on sex and gender	Not applicable.
Population characteristics	Not applicable.
Recruitment	Not applicable.
Ethics oversight	Not applicable.

Note that full information on the approval of the study protocol must also be provided in the manuscript.

Field-specific reporting

Please select the one below that is the best fit for your research. If you are not sure, read the appropriate sections before making your selection.

☒ Life sciences ☐ Behavioural & social sciences ☐ Ecological, evolutionary & environmental sciences

For a reference copy of the document with all sections, see nature.com/documents/nr-reporting-summary-flat.pdf

Life sciences study design

All studies must disclose on these points even when the disclosure is negative.

Sample size	Sample size was not predetermined by any statistic methods. Animal experiments were carried out with at least 5 mice per group. Technical triplicates from one of at least two independent experiments were performed to determine statistical significance. For sample size and methods for statistical analysis are described in figure legends of relevant figures.
Data exclusions	We did not exclude any data for the analysis.
Replication	Results of immunoblotting, qRT-PCR, flow cytometric analysis were from at least two or three biological replicates and described in the figure legends of corresponding figures. Three biological replicates were used for generating scRNA-seq . At least five mice were grouped for each treatment and the exact number were described in figure legends of corresponding figures. Each experiment was performed at least twice and all replications produced similar or equivalent results or phenotypes.
Randomization	Same number of mice were randomly chosen into distinct treatment groups.
Blinding	Investigators were blinded to randomly allocate mice into each treatment group.

Reporting for specific materials, systems and methods

We require information from authors about some types of materials, experimental systems and methods used in many studies. Here, indicate whether each material, system or method listed is relevant to your study. If you are not sure if a list item applies to your research, read the appropriate section before selecting a response.

Materials & experimental systems

n/a	Involved in the study	
☐	☒	Antibodies
☐	☒	Eukaryotic cell lines
☒	☐	Palaeontology and archaeology
☐	☒	Animals and other organisms
☒	☐	Clinical data
☒	☐	Dual use research of concern

Methods

n/a	Involved in the study	
☒	☐	ChIP-seq
☐	☒	Flow cytometry
☒	☐	MRI-based neuroimaging

Antibodies

Antibodies used	CD11c-APC (eBioscience, 17-0114-82); CD80-PE (eBioscience, 12-0801-82); CD86-PE-Cy7 (eBioscience, 25-0862-82); CD3-APC (eBioscience, 17-0031-82); CD4-FITC (eBioscience, 11-0041-82); CD8-PE (eBioscience, 12-0084-82); CD25-PerCP-Cy5.5 (eBioscience,

	45-0251-82);Foxp3-PE-Cy7 (eBioscience, 25-5773-80); CD44-PE-Cy7 (eBioscience, 25-0441-82); CD62L-PerCP-Cy5.5 (eBioscience, 45-0621-82); gD (Santa Cruz, 21719); DYKDDDDK-Tag (Abmart, 3P8); GAPDH (Abways, AB0037); IDO1 (Proteintech, 66528).
Validation	Antibodies used in this study were described in the websites of commercial suppliers or in the previous literatures.

Eukaryotic cell lines

Policy information about cell lines and Sex and Gender in Research

Cell line source(s)	Hepa1-6 and HEK293T cells were purchased from the American Type Culture Collection (ATCC). VX2, SMMC7721 and 4T1 cells were obtained from the Chinese Academy of Science (Shanghai, China). Vero cells were a gift from Jiahuai Han at Xiamen University.
Authentication	Cell lines were authenticated genetically by the providers and we indirectly verified their identity by their morphology, growth behavior or transcriptomic profiles.
Mycoplasma contamination	All cell lines were tested for mycoplasma contamination before their use in experiment. None of these cell lines are contaminated by mycoplasma.
Commonly misidentified lines (See ICLAC register)	No commonly misidentified lines was used.

Animals and other research organisms

Policy information about studies involving animals; ARRIVE guidelines recommended for reporting animal research, and Sex and Gender in Research

Laboratory animals	the C57BL/6 mice (female, 6-8 weeks) and New Zealand white rabbits were purchased from China Wushi, Inc. Shanghai, China. Animals were housed under specific pathogen free conditions in a temperature-controlled room.
Wild animals	No wild animals were used in this study.
Reporting on sex	Findings were not applied to only one sex.
Field-collected samples	No field-collected samples was used in this study.
Ethics oversight	All studies were approved and supervised by the Institutional Review Board (IRB) of Mengchao Hepatobiliary of Fujian Medical University.

Note that full information on the approval of the study protocol must also be provided in the manuscript.

Flow Cytometry

Plots

Confirm that:

☒ The axis labels state the marker and fluorochrome used (e.g. CD4-FITC).

☒ The axis scales are clearly visible. Include numbers along axes only for bottom left plot of group (a 'group' is an analysis of identical markers).

☒ All plots are contour plots with outliers or pseudocolor plots.

☒ A numerical value for number of cells or percentage (with statistics) is provided.

Methodology

Sample preparation	Methods for the preparation of cells and their source were described in figure legends and Materials and Method section. Sample preparation was described in Materials and Methods section
Instrument	BD FACS Verse
Software	FlowJo
Cell population abundance	Abundance of sorted cells were validated by overlapping them into pre-sorted cells using Flowjo software.
Gating strategy	All gates were set based on FMO (full-minus one) stains and isotype control antibodies after appropriate compensation using single-stained compensation controls.

☒ Tick this box to confirm that a figure exemplifying the gating strategy is provided in the Supplementary Information.
